# Another potential etiology for cardiac manifestation after snakebite

**DOI:** 10.1186/s41182-025-00709-5

**Published:** 2025-02-19

**Authors:** Yoshihiro Aoki, Chris Smith, Koya Ariyoshi

**Affiliations:** 1 Coordination Office for Emergency Medicine and International Response, Acute and Critical Care Center, Nagasaki University Hospital, Nagasaki, Japan; 2 School of Tropical Medicine and Global Health, Nagasaki University, Nagasaki, Japan; 3 Department of Clinical Research, Faculty of Infectious and Tropical Diseases, London School of Hygiene and Tropical Medicine, London, UK; 4 Department of Clinical Medicine, Institute of Tropical Medicine, Nagasaki University, Nagasaki, Japan

## Abstract

This letter discusses the possibility of Takotsubo cardiomyopathy (TTC) as an alternative diagnosis in a recently reported case of acute myocardial infarction following a hump-nosed viper bite. The patient’s presentation, including delayed chest tightness, elevated troponin, ECG changes, and normal coronary arteries, coupled with complete recovery within 3 months, strongly suggests TTC. Multiple case reports have documented the association between snakebites and TTC, with proposed pathophysiological mechanisms including sympathetic surge from pain and stress, direct cardiotoxic effects of venom, and inflammatory mediators during envenomation. The excessive catecholamine response may trigger transient cardiac dysfunction characteristic of TTC. Recognizing TTC as a potential complication of snakebites has important clinical implications, as its management and prognosis differ from acute coronary syndrome. Understanding this association may enhance diagnostic approaches and treatment strategies in similar cases, particularly when normal coronary arteries and complete cardiac recovery are observed.

We read with great interest the case report by Wanninayake et al. describing a rare case of acute myocardial infarction with heart failure following a hump-nosed viper bite [1]. While the authors provide a thorough analysis of potential mechanisms for cardiac dysfunction, we believe Takotsubo cardiomyopathy (TTC) warrants consideration as a crucial differential diagnosis in this case.

The clinical picture of chest tightness developing on day 3, elevated troponin, ECG changes, and normal coronary arteries on CT angiography is highly suggestive of TTC. This condition is characterized by transient left ventricular dysfunction often triggered by acute physical or emotional stress, and it may present with focal or atypical variants rather than the classic apical ballooning [2–4]. Although the current case demonstrated concordance between ECG findings and echocardiographic abnormalities, the potential focal dysfunction in TTC does not rule out a possible etiology of TTC in this case. Furthermore, complete recovery of cardiac function within 3 months aligns with the natural history of TTC, as highlighted by the International Takotsubo Registry [5]. While the authors appropriately investigated the case, advanced imaging such as cardiac magnetic resonance (CMR) can further distinguish TTC from myocarditis or infarction, although this may not be feasible in resource-limited settings.

Several reports underscore the association between snakebites and TTC. Murase et al. [6] and Van Rensburg et al. [7] documented TTC following envenomation, while Sunil et al. noted TTC in 1% of snakebite victims [8]. A comprehensive review by Mishra et al. identified multiple envenomation-triggered TTC cases [9]. Of particular interest is the recently reported case by Wijesinghe et al. describing TTC as part of the ATAK (adrenaline, Takotsubo, anaphylaxis, and Kounis hypersensitivity-associated syndrome) phenomenon following a cobra bite [10].

Regarding pathophysiology, TTC in snakebite victims can result from synergistic mechanisms. These include an acute sympathetic surge due to intense pain and stress, direct cardiotoxic effects of venom, and inflammatory mediators released during envenomation. Excess catecholamines may act on myocardial adrenergic receptors, leading to the transient cardiac dysfunction characteristic of TTC [9]. Recognizing these overlapping pathways is essential to consider TTC as a differential diagnosis when coronary arteries are normal, yet significant myocardial dysfunction and subsequent recovery are observed.

The evolution of clinical, laboratory, and imaging findings provides critical insights for distinguishing TTC from acute coronary syndrome (ACS), particularly in resource-limited settings where advanced cardiac imaging may not be available. Biomarker patterns often show distinct characteristics: while both conditions demonstrate troponin elevation, TTC typically shows a modest rise relative to the extent of wall motion abnormality, with rapid normalization within days [11]. In contrast, ACS generally exhibits higher peak troponin levels with a more gradual decline. Brain natriuretic peptide (BNP) and N-terminal proBNP (NT-proBNP) levels frequently show a disproportionate elevation in TTC compared to the degree of cardiac dysfunction [11]. While TTC typically presents with ST-segment elevation mimicking ACS, this case showed ST depression on admission—an atypical presentation that occurs in about 8% of TTC cases [5]. In typical cases, ECG evolution follows a characteristic pattern that may help differentiate TTC from ACS, with deep and widespread T-wave inversions developing within 24–72 h, accompanied by QT interval prolongation that persists during the sub-acute phase [12]. These changes typically normalize gradually over weeks to months, though T-wave inversions may persist longer than wall motion abnormalities.

Wall motion recovery patterns also differ significantly between TTC and ACS. TTC characteristically shows complete or near-complete resolution of wall motion abnormalities within 4–8 weeks, regardless of the initial variant pattern. This recovery occurs independently of coronary territories and may follow different temporal sequences in atypical variants. According to a comprehensive study by Ghadri et al., atypical TTC forms demonstrate complete recovery of wall motion abnormalities within the same timeframe as typical TTC, despite different initial patterns [13]. This finding is relevant to this case, which presented with an atypical reduced lateral wall motion pattern. The presence of biventricular involvement, as seen in the reported case, does not preclude a diagnosis of TTC, as variants affecting both ventricles have been well-documented [14]. These findings highlight the importance of considering TTC in cases with atypical presentations or biventricular involvement, as the characteristic recovery pattern remains a key diagnostic feature across variants.

Given these distinct progression patterns, serial evaluation of cardiac function, biomarkers, and ECG changes becomes crucial for retrospective diagnosis when advanced imaging is unavailable. This systematic approach to temporal changes can provide valuable diagnostic information, particularly in cases where the initial presentation deviates from classic patterns.

Understanding TTC as a potential complication of snakebites carries important implications for clinical practice. Its prognosis differs from that of acute coronary syndrome, and management strategies may need to be adjusted according to the unique pathophysiological features of TTC. In addition, requirements for long-term follow-up and patient counseling regarding recurrence risk differ substantially from those of true myocardial infarction. While the present case and past reports provide valuable insights into the cardiac manifestations following envenomation, continued consideration of TTC in a similar contexts may further enhance our diagnostic approach and clarify the complex mechanisms involved, particularly when coronary arteries are normal and complete recovery of cardiac function is observed.

## Data Availability

No datasets were generated or analysed during the current study.

## Abbreviations

ACS: Acute coronary syndrome
ATAK: Adrenaline, Takotsubo, anaphylaxis, and Kounis hypersensitivity-associated syndrome
CMR: Cardiac magnetic resonance imaging
CT: Computed tomography
ECG: Electrocardiogram
TTC: Takotsubo cardiomyopathy
